# A Portable Voice Assisted Smart Drug Delivery System with Automated Pill Dispensing for Dementia Patients

**DOI:** 10.1002/alz70861_108830

**Published:** 2025-12-23

**Authors:** Dalene Ephme Ben George, Shivani M, Harshini I, Allwyn S

**Affiliations:** ^1^ Sri Sivasubramaniya Nadar College Of Engineering, Chennai, Tamil Nadu India

## Abstract

**Background:**

Alzheimer’s Disease is caused due to brain atrophy that affects memory, thinking and behavior. Older population are the most affected ranging from mild, moderate to severe cases. Progression of the disease can be slowed and the symptoms can be managed with regular intake of medications prescribed by doctors. Dementia patients often face challenges in taking the correct medications on time and distinguishing the drugs prescribed. This leads to an increased burden to the caregivers and also catalyzes the progression of disease. We have designed a Portable Smart Drug Delivery System that aids patients in medication adherence by automatically dispensing the right drug at the right time.

**Method:**

The pills are loaded in different compartments of the system and the caregiver or physician can input the dosage details such as the time and amount of medication to be taken. A RTC is used for maintaining an accurate medication schedule. At the scheduled time, a ESP‐32 microcontroller receives a signal which triggers an audio alert such as a voice prompt that notifies the patient. Servo motors open the correct compartment as per the pre‐programmed dosage details. Load cell sensor measures the amount of pills dispensed and IR sensor checks if the pills are retrieved from the compartment. The medications taken or missed are recorded and the data is stored in a local memory and displayed via a LCD display. A small DC water pump is connected to a water container which when activated by a microcontroller via a relay module, dispenses water that runs for a few seconds before automatically turning off.

**Result:**

The portable smart drug delivery system is responsible for delivering the correct prescribed medication at the scheduled time. Integrated with audio alerts and automatic compartment opening reduces the missing, delay or wrong uptake of doses. The data stored in memory and displayed in LCD helps physicians during their regular checkups and caregivers are notified about the medication status of patients on a daily basis.

**Conclusion:**

This system can potentially increase the medication adherence, promote independence of dementia patients and reduce caregiver workload.

